# Mature IgM-expressing plasma cells sense antigen and develop competence for cytokine production upon antigenic challenge

**DOI:** 10.1038/ncomms13600

**Published:** 2016-12-07

**Authors:** Pascal Blanc, Ludovic Moro-Sibilot, Lucas Barthly, Ferdinand Jagot, Sébastien This, Simon de Bernard, Laurent Buffat, Sébastien Dussurgey, Renaud Colisson, Elias Hobeika, Thierry Fest, Morgan Taillardet, Olivier Thaunat, Antoine Sicard, Paul Mondière, Laurent Genestier, Stephen L. Nutt, Thierry Defrance

**Affiliations:** 1 CIRI, INSERM, U1111, Université Claude Bernard Lyon 1, CNRS, UMR5308, École Normale Supérieure de Lyon, Univ. Lyon, 21 Avenue Tony Garnier, 69007 Lyon, France; 2 AltraBio SAS, 30 rue Pre Gaudry, 69007 Lyon, France; 3 INSERM SFR Biosciences Gerland, UMS3444/US8, 50 Avenue Tony Garnier, 69007 Lyon, France; 4 eBioscience, An Affymetrix Company, 140 bis Rue de Rennes, 75006 Paris, France; 5 Institute of Immunology, University Hospital, Albert Einstein Allee 11, Ulm 89073, Germany; 6 INSERM, UMR917, F-35043 Rennes, France; 7 Pôle de Biologie, Centre Hospitalier Universitaire, 35033 Rennes, France; 8 Université de Rennes 1, F-35065 Rennes, France; 9 The Walter and Eliza Hall Institute of Medical Research, 1G Royal Parade, Parkville, Victoria 3052, Australia; 10 Department of Medical Biology, University of Melbourne, Parkville, Victoria 3010, Australia

## Abstract

Dogma holds that plasma cells, as opposed to B cells, cannot bind antigen because they have switched from expression of membrane-bound immunoglobulins (Ig) that constitute the B-cell receptor (BCR) to production of the secreted form of immunoglobulins. Here we compare the phenotypical and functional attributes of plasma cells generated by the T-cell-dependent and T-cell-independent forms of the hapten NP. We show that the nature of the secreted Ig isotype, rather than the chemical structure of the immunizing antigen, defines two functionally distinct populations of plasma cells. Fully mature IgM-expressing plasma cells resident in the bone marrow retain expression of a functional BCR, whereas their IgG^+^ counterparts do not. Antigen boost modifies the gene expression profile of IgM^+^ plasma cells and initiates a cytokine production program, characterized by upregulation of CCL5 and IL-10. Our results demonstrate that IgM-expressing plasma cells can sense antigen and acquire competence for cytokine production upon antigenic challenge.

## Abstract (web summary)

Plasma cells produce secreted antibodies and are thought to lack expression of the membrane-bound immunoglobulins that constitute B-cell receptors. Here the authors show that IgM-expressing plasma cells maintain B-cell receptor expression and initiate cytokine production following antigen stimulation.

The B-cell antigen receptor (BCR) is a multiprotein complex consisting of a membrane-bound immunoglobulin (Ig) molecule associated with the Igα/Igβ heterodimer, which functions as a signalling subunit. One of the most enduring paradigms in the field of B-cell biology holds that plasma cells (PC) have permanently switched-off expression of membrane-bound Ig molecules to produce their secreted version, i.e., antibodies (Abs). This paradigm is largely grounded in the demonstration that B cells and plasma cells identities are maintained by an opposing set of transcription activators and repressors. The two most prominent members of this genetic network are: (i) Pax5, one of the key element responsible for B cell commitment during hematopoiesis^1^ and (ii) B lymphocyte-induced maturation protein (Blimp-1), a transcriptional repressor usually referred to as the master regulator of plasma cells differentiation^2^,^3^. Blimp-1 promotes plasma cells differentiation largely through direct repression of Pax5 (ref. 4). Pax5 negatively regulates plasma cells differentiation both directly, through repression of XBP-1 (X-box binding protein-1), a transcriptional activator which controls the secretory machinery of plasma cells^5^ and indirectly, through its positive regulatory effect on BTB domain and CNC homolog 2 (Bach 2), a direct transcriptional repressor of Blimp-1 (ref. 6).

In spite of this, a careful examination of the literature reveals that plasma cells-bearing surface Igs have been previously described. In particular, Racine and colleagues have reported the existence in the bone marrow (BM), up to 100 days after *ehrlichial muris* infection, of a population of CD138^hi^ cells with an ambiguous plasma cell/plasmablast phenotype, that largely lack expression of B220 and CD19 but unexpectedly express high levels of sIgM and MHC class II (ref. 7). More recently, the group of G. Kelsoe published that BM antibody-secreting cells (ASCs) that produce natural polyreactive IgM Abs also express surface IgM (ref. 8). Finally, Pinto and colleagues have documented that human IgA and IgM-expressing plasma cells isolated from the gut *lamina propria* or the BM display a functional BCR while IgG-expressing plasma cells do not^9^.

We report here that BCR-expressing plasma cells can be generated by deliberate immunization with NP-dextran (the T cell-independent form of the hapten (4-hydroxy-3-nitrophenyl) acetyl) and reactivated *in vivo* by their nominal antigen. We establish that this unexpected feature for mature BM plasma cells is not determined by the chemical structure of the immunizing antigen, but is associated with expression of the IgM isotype that dominates the response to polysaccharidic antigen. Finally, we demonstrate that antigenic challenge *in vivo* modulates the gene expression profile of IgM^+^ BM plasma cells and initiates a cytokine production program characterized by upregulation of CCL5/RANTES and IL-10 expression. In conclusion, IgM-expressing mature plasma cells constitute a unique population with a dual plasma cells/B cell identity that shares with B cells the capacity to sense antigen, and which can behave as cytokine-producing cells upon antigenic challenge.

## Results

### BM ASCs induced by NP-dextran express surface Igs

**Figure 1:**
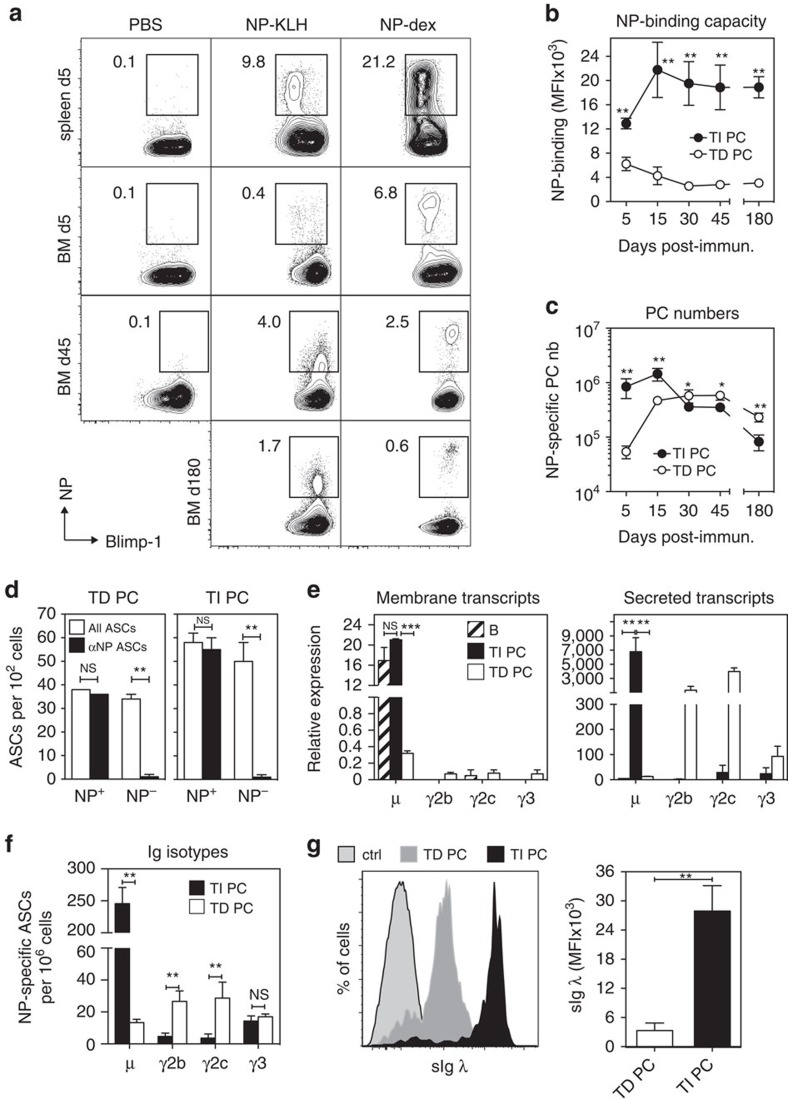
BM plasma cells induced by NP-dextran retain expression of membrane Igs. (**a**) NP-binding capacity of spleen and BM plasma cells after immunization with NP-KLH or NP-dextran. Numbers on the contour plots indicate the proportions of NP-specific plasma cells expressed as percentages of total BM plasma cells. (**b**) Compiled data illustrating the NP-binding capacity of TD and TI BM plasma cells at different time points after immunization. (**c**) Compiled data illustrating quantitation of the absolute numbers of BM NP-specific plasma cells at different time points after immunization. In **b** and **c**, data are expressed as means±s.d. of the values obtained in three independent experiments with three mice per time point. (**d**) Frequencies of polyclonal (all ASCs) and NP-specific ASCs assessed by ELISPOT in sorted NP^+^ and NP^−^ TD and TI BM plasma cells. Data are means±s.d. values of triplicate determinations gathered from three independent experiments in which NP^+^ and NP^−^ plasma cells were sorted from the pooled BM of five mice. (**e**) q-RT-PCR analysis of the membrane and secreted transcripts for the μ, γ2b, γ2c and γ3 Ig isotypes in B cells and sorted NP-specific TI and TD BM plasma cells. Results are expressed as relative quantity of mRNA after normalization to GAPDH expression. Data are means±s.d. of the values obtained in two independent experiments in which NP-specific plasma cells were sorted from pools of five immunized mice. (**f**) Frequency of BM ASCs producing IgM, IgG2b, IgG2c and IgG3 Abs after immunization with NP-KLH or NP-dextran. Data are means±s.d. of the values gathered from two independent experiments with three mice per group. (**g**) Density of expression of Ig lambda light chains by NP-specific TI and TD BM plasma cells. The bar chart on the right shows a compilation of the MFI values for Ig lambda stainings gathered from three independent experiments with three mice per group. The staining control was performed with an isotype-matched unrelated mAb. All experiments were conducted on *Blimp*^*gfp/+*^ mice. For experiments shown in **d**–**g**, plasma cells were recovered 45 days after immunization. NS: non significant, *P<0.05, **P<0.01 and ***P<0.001 (Mann–Whitney test). PC, plasma cells.

We and others, have documented that prototypic T-cell-independent (TI) antigen such as bacterial capsular polysaccharides can generate both effector and long-lived ‘memory' plasma cells residing in the BM like their T-cell-dependent (TD) counterparts^10^,^11^,^12^. Because, TI memory B cells have been demonstrated to be phenotypically and functionally distinct from conventional TD memory B cells^13^, we decided to explore whether this dichotomy also applies to memory plasma cells induced by these two types of antigen. We initially sought to compare the gene expression profile of early TD and TI plasma cells, also designated as plasmablasts (PB), generated by the same antigenic epitope (the hapten NP) conjugated either to a protein (NP-KLH) or to a polysaccharidic (dextran) carrier. PB being known to retain some expression of surface Igs^14^ we postulated that NP-specific PB could be identified thanks to the binding of the phycoerythrin (PE)-conjugated form of NP (NP-PE). We first analysed the binding of NP-PE by splenic PB of *Blimp*^*gfp/*+^ mice, five days after immunization. At this time point, TD and TI NP-specific PB (gated as CD138^hi^/B220^lo^/GFP^+^/NP-PE^+^ cells (Supplementary Fig. 1a) in the spleen display a comparable NP-binding capacity (Fig. 1a). However, analysis of NP-PE stainings on BM plasma cells at later time points (Fig. 1a,b) unexpectedly revealed that the NP-binding capacity of TI BM plasma cells increased between day 5 and day 15 and remained stable thereafter up until day 180 post-immunization. By contrast the NP-binding capacity of NP-specific TD BM plasma cells steadily declined between day 5 and day 30 and was consistently 6–8 fold lower than that of their TI counterparts at later time points (Fig. 1a,b). The numbers of NP-specific TI BM plasma cells reached a peak at day 15 post-immunization and declined thereafter to 8 × 10^4^ NP-specific plasma cells per mouse at day 180 post-immunization (Fig. 1c). This value falls within the range of the numbers of virus-specific ASCs recorded 150 days and more after LCMV infection^15^ NP-specific TD BM plasma cells were outnumbered by their TI counterparts at early time points after immunization, but were on average 3 fold more represented than NP-specific TI plasma cells at day 180 post-immunization. Analysis of the frequency of polyclonal ASCs (all ASCs) and NP-specific ASCs in sorted NP-PE^+^ and NP-PE^−^ BM plasma cells (see gatings in Supplementary Fig. 1b) demonstrated that both TD and TI NP-binding plasma cells are *bona fide* ASCs and that NP binding correlates with the ability of plasma cells to secrete NP-specific Abs (Fig. 1d). We then monitored the levels of expression of the membrane and secreted Ig heavy chain transcripts in TD and TI NP-specific plasma cells and splenic B cells by Q RT-PCR. Figure 1e shows that NP-specific TD plasma cells followed the commonly accepted rule, i.e., expressed high levels of the γ2b, γ2c and γ3 secreted transcripts while poorly expressing the membrane form of these transcripts. NP-specific TI plasma cells displayed a strikingly different profile inasmuch as: (i) they expressed high levels of both the secreted and the membrane form of the μ transcript (comparable to those expressed by B cells for the latter), (ii) the γ heavy chain secreted transcripts were only marginally represented. The heavy chain mRNA profiles of TD and TI NP-specific plasma cells were coherent with their pattern of Ig isotypes production (Fig. 1f) and with the fact that IgM is by far the dominant Ig isotype secreted in response to TI antigen. As shown in Fig. 1g, the density of expression of surface lambda light chains, the main Ig light chain isotype associated with anti-NP Abs^16^ followed the same trend as NP-binding, and was on average eight times lower on NP-specific TD plasma cells than on NP-specific TI plasma cells. Together, these findings demonstrate that NP-dextran immunization generates an atypical NP-specific plasma cells population that retains high expression of surface Igs (sIgs) up to 180 days after immunization.

### BM ASCs induced by NP-dextran exhibit a plasma cell identity

**Figure 2:**
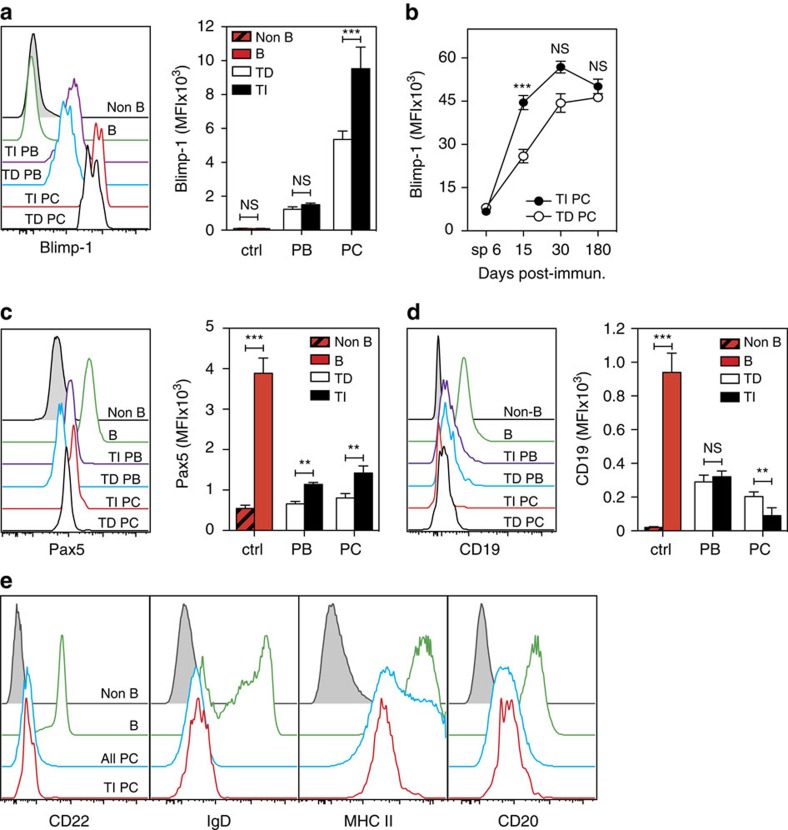
NP-specific BM ASCs induced by NP-dextran display a plasma cell identity. (**a**) Flow plots of Blimp-1/GFP (**a**), Pax5 (**c**) and CD19 (**d**) expression by splenic B cells, TD and TI splenic PB (6 days post-immunization), TD and TI BM plasma cells (30 days post-immunization). A compilation of the Blimp-1, Pax5 and CD19 MFI values is shown on the right of **a**,**c** and **d**. Cells lacking the B lineage and plasma cells markers (B220^−^/CD19^−^/GFP^−^, see gates in Supplementary Fig. 1b) were used as negative staining controls (gray-filled histograms). (**b**) Quantification of the Blimp-1 expression levels by NP-specific splenic PB (sp 6) and BM plasma cells at different time points after immunization. The bar charts in **a**,**c** and **d** and the graph shown in **b** illustrate the means±s.d. values gathered from two separate experiments each conducted with four individual mice. *P* values were calculated with the Mann–Whitney test. (**e**) Flowplots of CD22, IgD, MHCII I-A^b^ and CD20 expression by mature BM B cells, polyclonal BM plasma cells (all plasma cells) and NP-specific TI plasma cells, 30 days after NP-dextran immunization. Non-B/non-plasma cells cells (gray-filled histograms) were used as negative staining controls. All experiments were conducted with *Blimp*^*gfp/*+^ mice. PC, plasma cells.

To confirm that IgM^+^ BM plasma cells generated by NP-dextran display the hallmarks of plasma cells identity despite their expression of sIgs, we analysed by flow cytometry their expression of Blimp-1, Pax5 and CD19 (one of the direct targets of Pax5). For the sake of comparison, expression of these 3 markers was also analysed in: (i) splenic B cells (B-cell identity), (ii) BM plasma cells generated by NP-KLH (plasma cells identity), (iii) splenic PB induced by NP-dextran or NP-KLH. As shown in Fig. 2a, the levels of Blimp-1 expression in BM plasma cells are on average 5 (TD plasma cells) to 10 (TI plasma cells) times superior to those expressed by splenic PB. From day 30 post-immunization onwards, both NP-specific TI and TD plasma cells are Blimp-1^hi^ (Fig. 2b), a phenotypic trait known to be a distinctive feature of mature plasma cells^17^. In coherence with their pattern of Blimp-1 expression, both NP-specific TD and TI plasma cells display low levels of Pax5 and CD19 expression as compared with B cells but Pax5 is slightly more conserved in the latter plasma cells population than in the former (Fig. 2c,d). We next investigated whether TI NP-specific plasma cells conserved other phenotypical attributes of mature B cells, beyond expression of sIgs. Four markers were analysed for their expression by NP-specific TI plasma cells: CD20, a pan B cell surface molecule, IgD and CD22, both involved in modulation of BCR signalling and MHC class II molecules. In contrast to BM mature B cells, both polyclonal and NP-specific TI plasma cells expressed only marginal levels of IgD and CD22. As described earlier^18^ polyclonal BM plasma cells retained significant amounts of CD20 expression but at lower levels than mature B cells and NP-specific TI plasma cells followed the same trend. NP-specific TI plasma cells homogeneously expressed low but clearly detectable amounts of MHCII protein while expression of this marker on polyclonal plasma cells was more heterogeneous and divided this population into MHCII^lo^ and MHCII^hi^ subsets (Fig. 2e). Altogether these findings indicate that TI BM plasma cells like their TD counterparts exhibit the expected canonical phenotypic features of plasma cells, that is: high expression of Blimp-1 and low expression of Pax5. Interestingly they also display a partial B cell identity characterized by their strong expression of sIgs and moderate expression of MHC II proteins and CD20.

### BM ASCs induced by NP-dextran are mature plasma cells

**Figure 3:**
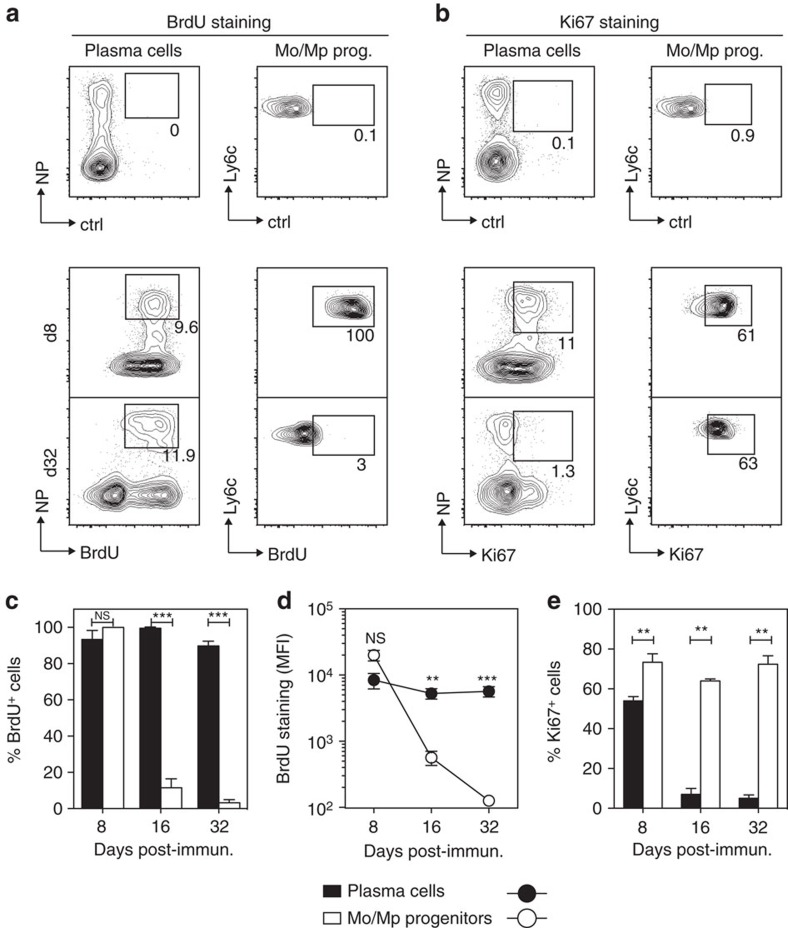
NP-specific BM plasma cells induced by NP-dextran are quiescent. BrdU (**a**) and Ki67 stainings (**b**) were conducted on NP-specific BM TI plasma cells and Mo/Mp progenitors (left and right contour plots, respectively) at day 8 and 32 after immunization of *Blimp*^*gfp/+*^ mice with NP-dextran. The staining control was performed with an isotype- and fluorochrome-matched unrelated mAb. Numbers shown on the contour plots indicate: (i) the % of NP-binding BrdU^+^ or Ki67^+^ plasma cells among the whole BM plasma cells population, (ii) the % of BrdU^+^ or Ki67^+^ Mo/Mp progenitors, (**c**) and (**e**) Compilations of the Ki67 and BrdU staining data. Results are expressed as % BrdU^+^ or Ki67^+^ cells among the NP-specific plasma cells and Mo/Mp progenitor populations. (**d**) Analysis of the staining intensity (MFI) of BrdU^+^ cells in the NP-specific plasma cells or Mo/Mp gates at different time points after immunization. Data shown in **c**,**d** and **e** represent the means±s.d. of the values gathered from two separate experiments conducted with four individual mice each. *P* values were calculated with the Mann–Whitney test.

Because of their poor biodegradability, polysaccharidic antigen may be retained in the organism for long periods of time^19^ leading to a prolonged wave of PB production. Although the high level of Blimp-1 expression by TI BM plasma cells was one element in favour of their maturity, it was of crucial importance to bring additional proof that the NP-binding ASCs we analysed in the BM after NP-dextran immunization fulfill the definition of mature plasma cells. PB, as opposed to mature plasma cells, are generally defined as actively dividing cells. We thus investigated the proliferative status of BM NP-specific TI plasma cells in *Blimp*^*gfp/*+^ mice at early (8 days) and late (32 days) time points after immunization, using both an *in vivo* BrdU pulse/chase strategy and Ki67 staining. The BM Ly6c^hi^/CD11b^+^ population enriched for monocyte (Mo) and macrophage (Mp) progenitors was used as a positive control in these experiments because these cells are subject to constant renewal in the BM. BM plasma cells and Mo/Mp progenitors were gated as illustrated in Supplementary Fig. 1d. All NP-specific BM plasma cells and Mo/Mp progenitors were BrdU-labelled at day 8 (Fig. 3a,c), indicating that both cell types had been actively dividing during the 8 days of the BrdU pulse. At day 32 post-immunization, Mo/Mp progenitors were mostly BrdU^−^ indicating that they had continued to divide during the chase period of the experiment. By contrast, all NP-specific plasma cells were still BrdU-labelled, suggesting that little subsequent division had occurred in this population during the 24 days that separated the arrest of BrdU feeding from BrdU staining analysis. The staining intensity of BrdU^+^ NP-specific plasma cells remained stable between day 16 and day 32 while it dropped dramatically for Mo/Mp progenitors within the same time period (Fig. 3d). This confirms that, after day 8 post-immunization, dilution of the plasma cells pool by new incomers is marginal. The pattern of Ki67 expression was consistent with the BrdU staining results inasmuch as both plasma cells and Mo/Mp progenitors were mostly Ki67^+^ at day 8 while only plasma cells became Ki67^−^ at day 32 post-immunization (Fig. 3b,e). Altogether, these results indicate that by day 32 post-immunization, NP-specific BM plasma cells have a slow turnover rate and have not been replaced by newly-formed plasma cells possibly generated from proliferating precursors during the BrdU chase period. Hence, from day 32 post-immunization onwards, NP-binding TI BM plasma cells constitute a mature plasma cells population. Since they also retain a high NP-binding capacity up to 180 days post-immunization (Fig. 1b), it is unlikely that they originate from a long-lasting ongoing TI immune response.

### BM plasma cells induced by NP-dextran express a functional BCR

**Figure 4:**
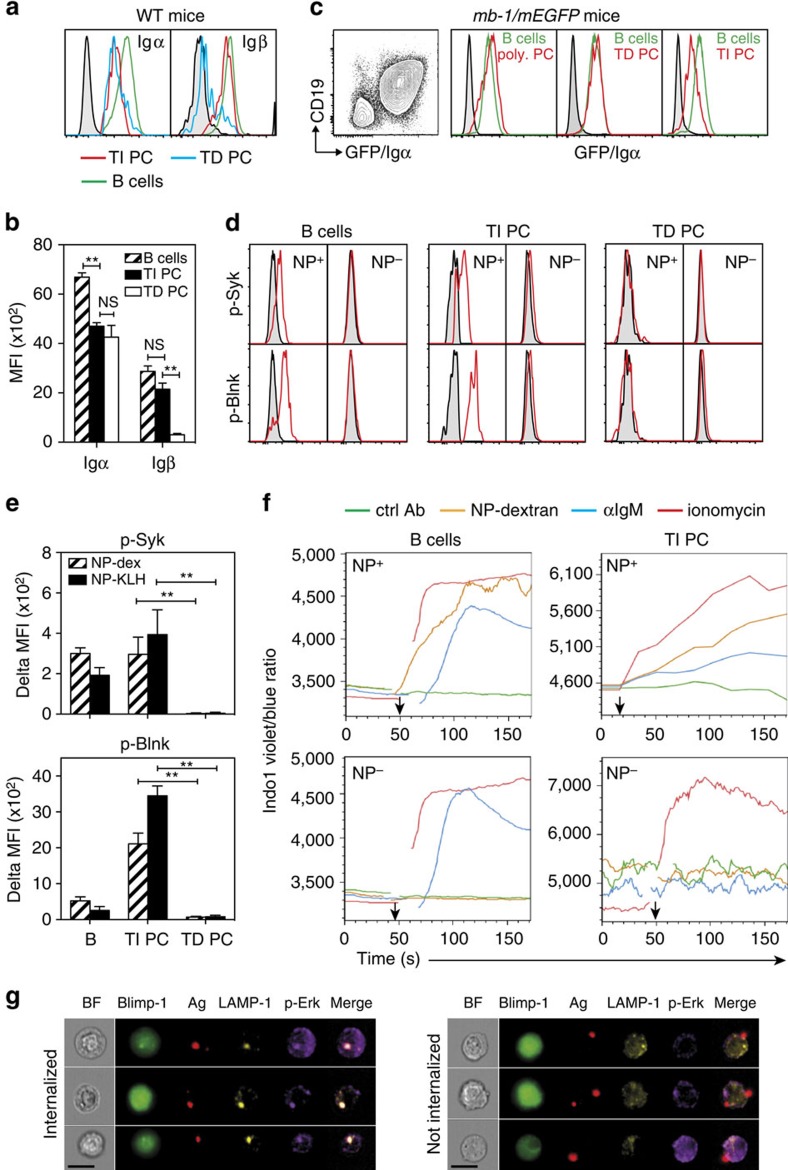
NP-specific T-cell-independent BM plasma cells express a functional BCR. (**a**) Expression of Igα and Igβ by splenic B cells and NP-specific BM plasma cells 45 days after NP-KLH or NP-dextran immunization. (**b**) Compilation of the MFI values of the Igα and Igβ stainings shown in **a**. (**c**) Pattern of Igα expression by plasma cells using Igα-reporter mice, 70 days after NP-dextran or NP-KLH immunization. From left to right: expression of GFP/Igα by enriched BM plasma cells cells, polyclonal plasma cells, NP-specific TD and TI plasma cells, respectively. (**d**) BCR-induced phosphorylation of Syk (upper panels) and Blnk (lower panels) in NP-binding (NP^+^) and non NP-binding (NP^−^) B cells, NP-specific TI and TD BM plasma cells after *in vitro* stimulation with NP-dextran. (**e**) Compilation of the delta MFI values for Syk (top) and Blnk (bottom) after *in vitro* stimulation with NP-dextran or NP-KLH. (**f**) Ca^++^ mobilization induced by BCR ligation in B cells (left) or TI BM plasma cells (right) after *in vitro* culture with the indicated stimuli. Graphs illustrate Indo 1 fluorescence traces versus time. The *y* axis shows the indo-1 violet to blue fluorescence ratio, an indicator of intracellular Ca^++^ levels. (**g**) BM plasma cells were enriched from *Blimp*^*gfp/+*^ mice 45 days after immunization with NP-dextran, incubated with flash red-conjugated polystyrene beads coated with NP-OVA and stained with LAMP-1 and p-Erk-specific mAbs after permeabilization. Cells were analysed by epifluorescence microscopy in flow (scale bar: 10 μM). The figure depicts representative images of plasma cells that have (left panel) or have not (right panel) internalized antigen. BF: brightfield. Data in **b**–**e** represent the means±s.d. of the values gathered from three separate experiments conducted with three mice each. *P* values were calculated with the Mann–Whitney test. Data in **f**–**g** are representative of two experiments conducted with plasma cells enriched from pools of four mice. Staining controls (gray-filled histograms): isotype-matched unrelated mAb (**a**), non-B/non plasma cells (**c**), unstimulated cells (**d**). Mice: C57Bl/6 (**a**), *mb-1/mEGFP* heterozygous mice (**c**), QM X C57Bl/6 F1 mice (B cells in **d**,**f**), *Blimp*^*gfp/+*^ mice (TD and TI plasma cells in **d**,**g**), QM X *Blimp*^*gfp/+*^ mice (plasma cells in **f**). PC, plasma cells.

To determine whether membrane-bound Igs expressed by TI BM plasma cells are signalling-competent, we first analysed expression of the Igα/Igβ heterodimer on these cells. As shown in Fig. 4a (left panel), NP-specific TD and TI BM plasma cells expressed comparable levels of Igα, that were on average reduced by 40% as compared with B cells (Fig. 4b). Because it recognizes an intra-cytoplasmic epitope of the molecule, our anti-Igα mAb did not allow us to determine which fraction of the Igα pool is located at the plasma membrane in plasma cells. This could be achieved for Igβ because the mAb used for staining binds an extracellular epitope of the molecule. As shown in Fig. 4a (right panel) and Fig. 4b, NP-specific TD plasma cells almost lacked surface Igβ expression while the levels of expression of Igβ by their TI counterparts was comparable to that of B cells. Analysis of *mb-1/mEGFP* heterozygous mice^20^ expressing mEGFP from one of the two mb-1 (Igα) alleles confirmed that Igα expression is maintained in polyclonal BM plasma cells as well as in TI and TD NP-specific BM plasma cells (Fig. 4c). We next examined whether ligation of the BCR expressed by TI plasma cells could induce phosphorylation of the Syk kinase and its substrate, the adaptor molecule Blnk/SLP65, that are both receptor-proximal signal transducer elements of the BCR. The gates used for the phospho flow assays are shown in Supplementary Fig. 1e. We observed that *ex vivo* stimulation of B cells and TI plasma cells with NP-dextran induced phosphorylation of Syk and Blnk in NP-binding cells but not in non NP-binding cells. By contrast, TD plasma cells (regardless of their capacity to bind NP-PE) showed very little detectable p-Blnk and virtually no p-Syk staining upon stimulation with NP-dextran (Fig. 4d) or NP-KLH (Fig. 4e).

To further document the signalling ability of the BCR expressed by NP-specific TI plasma cells, we examined its capacity to mobilize Ca^++^ after *in vitro* ligation. Gatings for the Ca^++^ mobilization assays are shown in Supplementary Fig. 1f. As illustrated by Fig. 4f, prior labelling with NP-PE did not prevent NP^+^ B cells from mobilizing Ca^++^ in response to either NP-dextran, goat anti-IgM Abs or ionomycin. NP^−^ B cells responded well to the surrogate antigen and ionomycin but, as expected, did not raise their intracellular Ca^++^ levels in response to NP-dextran stimulation. No Ca^++^ signal above threshold was observed in any of the cell population analysed in response to the control Ab. *In vitro* stimulation with ionomycin, NP-dextran and anti-IgM Abs but not with the control Ab also induced Ca^++^ mobilization in NP^+^ TI BM plasma cells. Conversely, only ionomycin was able to significantly induce Ca^++^ mobilization in NP^−^ TI BM plasma cells.

Finally, TI plasma cells were incubated with APC-conjugated polystyrene beads coated with NP-OVA and subsequently stained with Abs against LAMP-1 (to visualize late endosomes) and phospho-Erk, a downstream kinase mostly activated in the endosomes^21^ to monitor antigen internalization. Cells were next examined for co-localization of antigen-coated beads, LAMP-1 and p-Erk (Fig. 4g). On average, 6% of the analysed GFP^+^ plasma cells exhibited internalized antigen particles for which the red fluorescence of the antigen-coated beads co-localized with the LAMP-1 and p-Erk stainings hence generating a bright signal. This percentage is coherent with the representation of NP-specific plasma cells amongst the enriched BM plasma cells population (5–10% on average). Collectively, the aforementioned observations provide evidence that the BCR expressed by TI BM plasma cells is signalling-competent and promotes both the early and late events of the BCR signalling cascade when it is engaged.

### BCR-expressing BM plasma cells are IgM+

**Figure 5:**
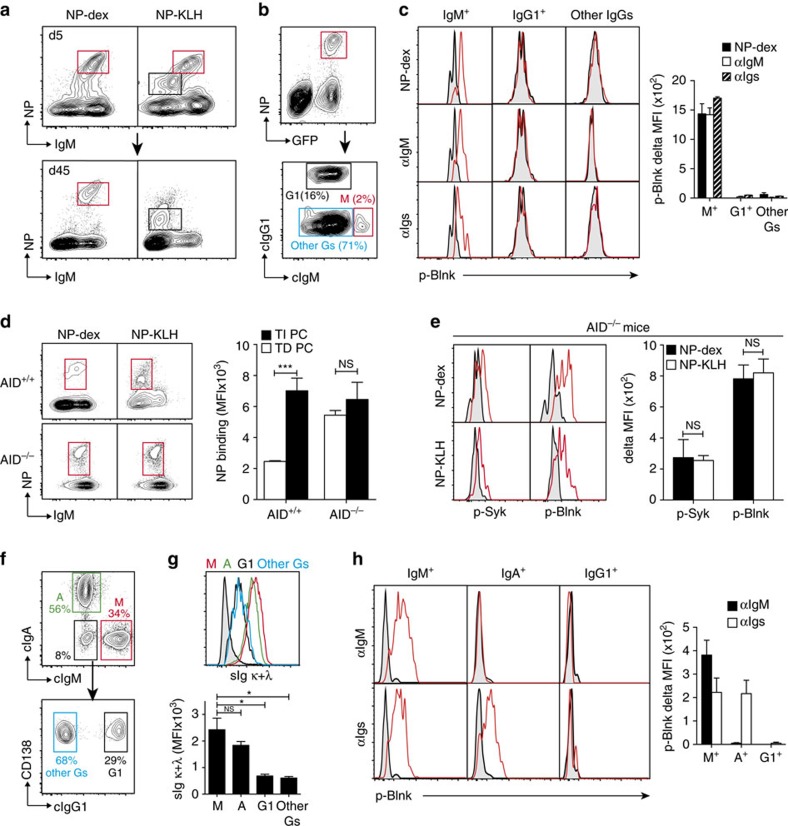
Maintenance of the BCR on plasma cells is associated with IgM expression. (**a**) sIgM expression by TD and TI NP-specific splenic PB (day 5) and BM plasma cells. Low and high NP-binding cells are gated in black and red, respectively. (**b**) Intracytoplasmic staining of BM TD plasma cells with NP-PE (top) and subdivision of NP-specific plasma cells according to cytoplasmic expression of IgG1 and IgM (bottom). (**c**) Patterns of Blnk phosphorylation in IgM^+^ and IgM^−^ NP-specific TD plasma cells in response to NP-dextran, anti-IgM or anti-Igs F(ab′)2 Abs (red histograms). On the right, compilation of the delta MFI values for the stainings displayed on the left. (**d**) NP-binding capacity of TD or TI BM plasma cells recovered from AID^+/+^ or AID^−/−^ mice. On the right, compilation of the NP-binding MFI values for the stainings displayed on the left. (**e**) Syk and Blnk phosphorylation profiles of NP-specific TD BM plasma cells recovered from AID^−/−^ mice, upon *in vitro* stimulation with NP-dextran (top) or NP-KLH (bottom). On the right, compilation of the delta MFI values for the stainings displayed on the left. (**f**) Enriched polyclonal BM plasma cells from naïve mice were stained for intracytoplasmic IgM, IgA and IgG1. (**g**) (top) Expression of kappa/lambda Ig light chains in the four plasma cells populations gated in **f**. The bar chart (bottom) shows a compilation of the MFI values for the kappa/lambda stainings displayed in the top panel. (**h**) Enriched polyclonal BM plasma cells from naïve mice were stimulated *ex vivo* with anti-IgM or anti-Igs F(ab')2 Abs and stained with anti-IgM, IgA, IgG1 and p-Blnk mAbs (red histograms). Plasma cells were recovered 45 days after immunization in all experiments. Data in **c**, **d** and **e** represent the means±s.d. of the values gathered from three separate experiments conducted with three mice each. Data in **g** and **h** represent the means±s.d. of the values gathered from two separate experiments conducted with four mice each. *P* values were calculated with the Mann-Whitney test. Mice: C57Bl/6 (**f**–**h**), *Blimp*^*gfp/+*^ (**a**,**b**), AID ^−/−^ (**e**). Staining controls (gray-filled histograms): unstimulated cells (**c**,**e**,**h**), isotype-matched unrelated control mAb (**g**). PC, plasma cells.

In the mouse, IgM-expressing plasma cells are prominent among the memory plasma cells compartment elicited by polysaccharidic antigen with only a minor contribution of switched plasma cells belonging to the IgG3 or IgA isotypes^10^,^11^. Conversely, when administered subcutaneously, TD antigen mainly give rise to IgG-secreting memory plasma cells and only to a few long-lived IgM-expressing plasma cells. The pattern of Ig isotype expression by NP-specific TI and TD plasma cells was in accordance with this paradigm inasmuch as the former were mostly IgM^+^ while the latter were predominantly IgM^−^ (Fig. 1e,f). Interestingly, NP-PE stainings conducted on early TD PB revealed two intensities of NP-binding on these cells: high for IgM^+^ PB, low for IgM^−^ PB (Fig. 5a). This finding led us to examine the possible relationship between the nature of the Ig isotype produced by plasma cells and their expression of a functional BCR. Intracytoplasmic staining of TD BM plasma cells with NP-PE, anti-IgG1 and anti-IgM mAbs discriminated three NP-specific plasma cells populations: a small but detectable IgM-expressing subset, a substantial IgG1-expressing plasma cells compartment and a large population of plasma cells expressing neither IgM nor IgG1 (Fig. 5b, lower panel). Because subcutaneous NP-KLH immunization does not generate any detectable NP-specific IgA^+^ plasma cells in the BM, the double negative plasma cells population most likely consists of NP-specific ASCs expressing IgG subclasses other than γ1 (γ2b, γ2c and γ3). As illustrated by Fig. 5c, NP-dextran as well as surrogate antigens induce Blnk phosphorylation in IgM-expressing NP-specific TD plasma cells but not in the IgG-expressing ones.

To further explore the link between Ig isotype expression and maintenance of a functional BCR on plasma cells, we switched to AID^−/−^ mice. Due to invalidation of the AID gene that controls the class switch recombination and somatic hyper mutation processes, these mice produce only unmutated IgM Abs upon immunization. As illustrated by Fig. 5d,e, TD and TI plasma cells generated in AID^−/−^ mice exhibited comparable levels of NP-binding. By contrast, in AID^+/+^ control littermates, high levels of NP-binding were maintained for TI plasma cells only. The BCR expressed by NP-specific TD plasma cells generated in AID^−/−^ mice was fully functional as demonstrated by its ability to promote Blnk phosphorylation upon *in vitro* triggering by NP-dextran or NP-KLH (Fig. 5e).

Finally, as another approach to document the influence of the Ig isotype on maintenance of a functional BCR on plasma cells, we next compared the levels of expression of surface Ig light chains and the signalling capacity of the BCR for IgM-, IgA- and IgG-expressing polyclonal BM plasma cells at the steady-state. As shown in Fig. 5f, four main populations could be discriminated within the polyclonal BM plasma cells compartment, based on their cytoplasmic Ig isotype content: (i) cIgM^+^, (ii) cIgA^+^, (iii) cIgG1^+^ and (iv) a composite cIgM^−^/cIgA^−^/cIgG1^−^ subset likely including cIgG2b/c^+^ and cIgG3^+^ plasma cells. Surface Ig light chains were expressed at high density on both cIgM^+^ and cIgA^+^ plasma cells while their level of expression was reduced by 70–75% on cIgG^+^ plasma cells (Fig. 5g). As shown in Fig. 5h, both anti-Ig (H+L) and anti-IgM Abs promoted Blnk phosphorylation in cIgM^+^ plasma cells while cIgA^+^ plasma cells were only responsive to anti-Ig Abs (H+L). By contrast, cIgG1^+^ plasma cells failed to respond to any of these surrogate antigen. Taken together, these findings demonstrate that expression of a functional BCR by immunization-induced BM plasma cells is not dictated by the chemical structure of the antigen but is linked with expression of the IgM isotype. They also suggest that maintenance of BCR expression could be shared by IgM and IgA-expressing plasma cells.

### Antigen stimulation drives IgM^+^ BM plasma cells to cytokine production

**Figure 6:**
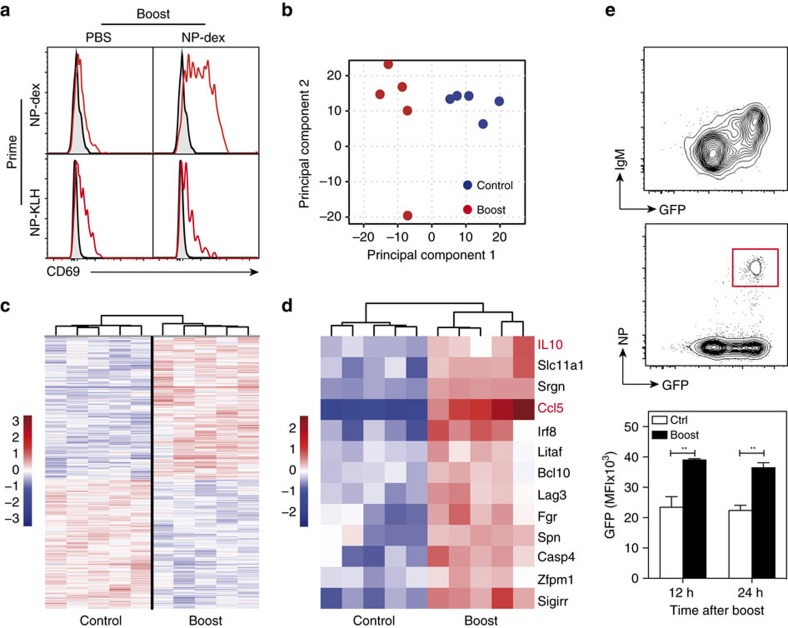
**Antigen challenge modifies the gene expression profile of IgM**^**+**^ **BM plasma cells.** (**a**) *Blimp*^*gfp/+*^ mice were primed sc with NP-dextran or NP-KLH and boosted iv at day 60 with NP-dextran (boost) or injected with PBS (control). NP-specific BM plasma cells were analysed 12 h after secondary immunization for CD69 expression (red histograms). Staining profiles obtained with the isotype-matched unrelated control mAb are depicted as gray-filled histograms. (**b**) Principal component analysis (PCA) of the gene expression profiles of unstimulated (control) and antigen-stimulated (boost) NP-specific TI BM plasma cells. The two groups of experimental samples (*n*=5 for each group) are projected on the first two principal components that account for 33.4% of the variance. (**c**) Dendrogram of the 1,000 most differentially-expressed genes between unstimulated (control) and antigen-stimulated (boost) NP-specific IgM^+^ BM plasma cells. The analysis has been conducted on five distinct samples for each experimental group. (**d**) Heatmap of the 13 most significantly differentially-expressed genes associated with the GO terms: cytokine production and regulation of cytokine production. (**e**) Impact of antigen stimulation on GFP/IL-10 expression by NP-specific TI plasma cells from IL-10 reporter Vert-X mice. Vert-X mice were injected iv with PBS 45 days after priming with NP-dextran. IgM-expressing plasma cells (left) and NP-binding plasma cells (right) BM plasma cells were analysed for their expression of GFP. Plasma cells were gated as CD138^hi^/Ly6c^+^ cells as shown in Supplementary Fig. 1c. Representative of two experiments conducted with four mice each. The bar charts show a compilation of the GFP MFI values for NP-specific plasma cells 12 h after NP-dextran boost *in vivo* or injection of PBS boosted (boost) or not (ctrl) with NP-dextran *in vivo*. Data represent the means±s.d. of the values gathered from two separate experiments with four mice per group.

To determine whether IgM^+^ BM plasma cells can be activated by antigen *in vivo*, we first monitored induction of the early activation marker CD69 upon antigenic challenge. As shown in Fig. 6a, CD69 was expressed by most NP-specific TI plasma cells after antigen exposure *in vivo* but not by unstimulated plasma cells. No significant expression of CD69 was found on NP-specific TD plasma cells in both the control and antigen-challenged groups, even when NP-KLH was substituted to NP-dextran for the boost (data not shown). Assessment of CD69 expression on TD plasma cells at earlier (6 h) and later (24 and 36 h) time points after boost established that their lack of CD69 upregulation upon antigen stimulation is not due to a precipitated or delayed activation process in these cells (data not shown).

We next investigated the impact of *in vivo* antigen stimulation on the gene expression profile of IgM^+^ BM plasma cells using Affimetrix GeneChip cDNA microarrays. As illustrated by Fig. 6b, the first principal component separated unstimulated (control) plasma cells from antigen-stimulated plasma cells (boost) and clearly restituted our experimental design. This result, together with the heatmap illustrating the 1000 most significantly modulated genes upon antigen boost (Fig. 6c; Supplementary Data 1) demonstrates that antigenic stimulation profoundly modifies the gene expression profile of IgM^+^ BM plasma cells. As shown in Table 1, the Ccl5/RANTES mRNA was the most highly modulated transcript upon antigen stimulation of IgM^+^ plasma cells (fold change: 8.6, *q* value: 0.0014). To determine the biological themes to which the modulated genes displayed in Table 1 are related to, the 20 genes with the highest absolute fold change and adjusted *P* value <0.05 were tested for enrichment of GO terms using the MGSA Bayesian approach^22^. As shown in Table 2, the biological processes related to regulation of cytokine production and cytokine production were the two most affected gene sets in the antigen-stimulated plasma cells group.

The heatmap of the 13 most significantly modulated genes belonging to the two GO terms related to cytokine production (Fig. 6d) highlights IL-10 as another cytokine that may be subjected to upregulation in IgM^+^ plasma cells upon antigen stimulation (fold change: 1.7, *q* value 0.026). To confirm the impact of antigen stimulation on IL-10 production by IgM^+^ plasma cells, we monitored its expression by NP-specific plasma cells in immunized IL-10 reporter (Vert-X) mice. In agreement with the original description of the Vert-X mouse by Madan and colleagues^23^, we found predominance of GFP expression in plasma cells with virtually no GFP expression in the other cell types constituting the CD138-enriched population (data not shown). Somehow surprisingly, constitutive transcription of the IL-10 gene within the polyclonal BM plasma cells population at the steady-state, is largely restricted to IgM^+^ plasma cells, independently of their ability to bind NP-PE (Fig. 6e). In accordance with the transcriptoma data, antigen boost *in vivo* induced an approximate twofold enhancement of the GFP reporter signal in NP-specific plasma cells (Fig. 6e). Altogether these findings suggest that BCR triggering on IgM^+^ plasma cells modifies their gene expression profile and potentiates their Ab-independent function of cytokine secretion.

## Discussion

Our present description of BCR-expressing plasma cells is iconoclastic with regard to the widely accepted rule that B cell and plasma cells identities are defined by a set of mutually opposing transcription factors^1^,^2^,^3^,^4^,^5^,^6^. We endeavoured to rule out any trivial explanation that may account for this unexpected observation. First, we have excluded the possibility that we might be dealing with cytophilic Igs produced and re-captured by plasma cells though Ig Fc receptors for example. Surface Igs expressed by IgM^+^ plasma cells are resistant to mild acidic treatment (data not shown) but more importantly, they can transduce a signal that activates some of the recognized components of the BCR signalling pathway (Syk, Blnk and Erk). Our demonstration that IgM^+^ plasma cells, in contrast to their IgG^+^ counterparts, express high levels of both the membrane and secreted forms of the Ig transcripts further supports the notion that sIgs are endogenously produced by IgM^+^ plasma cells. Second, we have excluded that BCR-expressing plasma cells could represent an early stage of plasma cells differentiation because they exhibit none of the expected features of PB. They are non-proliferating and their pattern of Pax5, CD19 and Blimp-1 expression is fully coherent with that of mature plasma cells and not with that of PB. The long-term persistence of a pool of antigen-specific plasma cells in the BM does not necessarily infer that these plasma cells are long-lived. Chernova and colleagues recently reported that an important fraction of polyclonal BM plasma cells and NP-specific plasma cells generated by NP-CγG immunization, characterized by expression of B220 and intermediate levels of Blimp-1, is short-lived and actively replenished by proliferative precursors^24^. However, antigen-driven replenishment is unlikely to account for the long-term persistence of IgM^+^ plasma cells generated in response to NP-dextran since they are still detectable 6 months after immunization, while the half-life of polysaccharides in the body has been estimated to be within the range of 8.5 to 63 days^19^. Furthermore, in contrast with the BM plasma cells population with a high turnover described by Chernova *et al*., the BCR-expressing IgM^+^ plasma cells we studied here lack B220 expression (data not shown) and express high levels of Blimp-1. Regarding that immunization with dextran (the polysaccharidic carrier of the immunogen used in our study) induces a population of long-lived, resting, IgM-secreting BM plasma cells resistant to a cyclophosphamide treatment^11^, the possibility that NP-specific IgM^+^ plasma cells induced by NP-dextran are long-lived is worth considering.

Finally, the atypical plasma cells population we describe here is not a singularity of the mouse model we are using. Its human counterpart also exists, as documented by Pinto and colleagues who reported that human IgA and IgM-expressing polyclonal plasma cells isolated from the gut lamina propria or the BM display a functional BCR while IgG-expressing plasma cells do not^9^. In keeping with this, it is noteworthy that IgM^+^BCR^+^ plasma cells present striking phenotypical and functional similarities with the tumoural plasma cells responsible for Waldenström macroglobulinemia (WM) disease. Like WM cells, they express IgM and carry a functional BCR^25^, and have the propensity to produce CCL5 (ref. 26). This point certainly deserves further investigation.

Interestingly, our data show that IgG^+^ plasma cells retain some antigen-binding capacity, in agreement with the earlier studies of Smith *et al*.^27^ and Cassese *et al*.^28^, as well as expression of Igα. The low antigen-binding capacity of IgG^+^ plasma cells (Fig. 1a,b) is the most obvious explanation to their unresponsiveness to antigen triggering but it must be balanced with the fact that IgG is also able to elicit stronger responses than IgM, at least partly due to the additional signalling motifs of its cytoplasmic tail^29^. Because increased activity of phosphatases such as SHP-1 and SHIP, known to regulate BCR signalling, has been described to impair responsiveness of germinal centre^30^ or anergic^31^ B cells to antigenic stimulation, the possibility of an active silencing of the signalling function of IgG BCR in plasma cells cannot be excluded. Conversely, the loss of IgD and CD22 expression by IgM^+^ plasma cells suggest that they could display an enhanced susceptibility to antigenic stimuli. Indeed, IgD has been described to dampen BCR signalling in response to monovalent antigen^32^ while CD22 is a known negative regulator of BCR signalling. This suggests that the overall threshold for full activation of IgM^+^ plasma cells in response to antigen stimulation might be lowered as compared with naive B lymphocytes. Together with their downregulated expression of CD19, these findings suggest that IgM^+^ plasma cells may be sensitive to activation by low-valence, uncomplexed antigen.

It remains that maintenance of Igα expression by IgM^+^ plasma cells is puzzling because it should be part of the plasma cells-inappropriate genes indirectly repressed by Blimp-1 through its negative regulatory effect on Pax5. High Blimp-1 expression by IgM^+^ BM plasma cells was associated with strong downregulation of Pax5 but surprisingly, although Igα and CD19 are both direct Pax5 targets^33^, only CD19 was strongly repressed in these cells. The use of Igα-reporter mice confirmed the stainings conducted with Abs and established that this BCR-associated signalling protein is effectively expressed by IgM^+^ plasma cells. This observation agrees with the recently published transcriptomics data of Wei Shi and colleagues demonstrating that there is only a twofold reduction in the Igα and Igβ transcript levels in BM plasma cells (as compared with follicular B cells) while CD19 goes down 16-fold in plasma cells^34^. The reason for the contrasting behaviour of CD19 and Igα with regard to Pax5 repression in IgM^+^ plasma cells is presently unclear but it points towards the possibility that chromatin remodelling could also be involved in maintaining expression of B cell-specific genes in these cells. In the same line of thought, the strong expression of the membrane-bound form of Igs by IgM^+^ plasma cells is another example of inappropriate maintenance of a B cell-specific feature by IgM^+^ plasma cells. The pre mRNA for immunoglobulin heavy chains is alternatively processed to produce the membrane or secreted mRNAs. The transcript shift from the membrane-bound to the secreted form of Igs is largely governed by the transcription elongation factor ELL2 and its associated factor EAF2 (ref. 35) which are both upregulated in plasma cells but poorly expressed in B cells^34^,^36^. Induction of these two factors could possibly be differently regulated in IgM^+^ and IgG^+^ plasma cells. The partial B cell identity displayed by IgM^+^ plasma cells extends beyond expression of the BCR and its co-receptors Igα and Igβ because we confirm here that they also express significant levels of CD20 and MHCII, as previously described^18^,^37^.

Altogether the observations outlined above suggest that IgM^+^ plasma cells with a dual B cell/plasma cells identity originate from a unique developmental program. What could be the driving force that governs generation of cells with such a split personality? Our data demonstrate that the chemical structure of the immunizing antigen can be ruled out because both the TD and TI forms of NP generate IgM^+^ plasma cells bearing a functional BCR in WT and AID^−/−^ mice. The possibility that they originate from a different mature B cell precursor than conventional plasma cells is also unlikely. NP-KLH immunization induces BCR-expressing IgM^+^ plasma cells at high and low frequencies in AID^−/−^ and WT mice respectively, despite the fact that the B cell subtypes recruited by NP-KLH are expected to be the same in both strains of mice. Our data rather suggest that programmatic differences initiated at the time of IgG class switching are responsible for expression of a BCR by plasma cells. It has recently been documented that the influence of the B cell transcriptional regulators involved in IgG2a (T-bet) and IgA (RORα) class-switch recombination extends beyond the sole expression of a class-switched BCR. They also lead to imprinting of molecular programs critical for maintenance of the identity, antigen-binding capacity and survival of memory B cells^38^. Interestingly, IgG (and IgE) isotypes differ from other Ig heavy chains by the presence of a long cytoplasmic tail (28 AA) containing an Ig tail tyrosine (ITT) signalling motif^29^. Because, IgG and IgM-containing BCRs have been documented to activate different sets of genes^39^ the ITT module could contribute to initiate a transcriptional program that will ultimately lead to downregulation of BCR expression by plasma cells.

Upon antigen recognition, IgM^+^ plasma cells initiate a biological response biased towards cytokine production, characterized in particular by upregulation of CCL5 and IL-10. This observation is in line with the growing body of evidence that designates plasma cells as a major cell source of cytokine production in the B lineage^40^. The current view of the cytokine-producing function of plasma cells is that it is initiated in B cells through the triggering of innate receptors. Our present work uncovers a novel mechanism whereby mature plasma cells may also be directly driven to cytokine production by antigenic stimuli in the course of a recall response. We can only speculate on the biological functions fulfilled by the cytokines produced by IgM^+^ plasma cells upon antigen recall. CCL5 is a chemokine that targets multiple cell types among which: T cells, dendritic cells, NK cells, mast cells, basophils and eosinophils^41^. Upregulation of its transcript in antigen-stimulated IgM^+^ plasma cells evokes the possibility that plasma cells-derived CCL5 could be important for alerting other immune cell partners about an infection. This antigen-induced chemokine-driven guidance could help to foster cellular interactions in a single microanatomic site or to favour communication between plasma cells and innate or adaptive effectors of the immune system. IL-10-mediated immunoregulation is one of the most documented non-canonical (i.e., unrelated to Ig secretion) biological function assigned to plasma cells^42^,^43^. One possible scenario is that IL-10 production by activated IgM^+^ plasma cells combined with their ability to produce CCL5 could allow these cells to dampen exaggerated and potentially harmful activation of innate effectors of the immune system, in the BM. It is noteworthy that the BM can function as a secondary lymphoid organ, especially in the course of infection by blood-borne microbes such as *S. typhimurium* that have a known tropism for the BM. In keeping with this, Cariappa and colleagues have reported that B cells can be activated in the BM and differentiate *in situ* in a TI manner into IgM-secreting cells in response to IV injection of *S. typhimurium*^44^.

BCR expression could also confer a survival advantage to IgM^+^ plasma cells, independently of antigen recognition. This form of antigen-independent survival signalling, referred to as ‘tonic' BCR signalling^45^, has been demonstrated to be instrumental for maintaining viability of mature peripheral B cells^46^,^47^. Because of the limited capacity of the plasma cells survival niches in the BM, the pool of established long-lived plasma cells is subject to downsizing to accommodate newly generated plasma cells with novel antigenic specificities. It has been proposed that IgG-containing immune complexes produced in response to novel antigenic challenges contribute to this regulation by causing apoptosis of memory plasma cells through engagement of their FcγRIIb^48^. In this context, tonic signals channeled through the BCR of IgM^+^ plasma cells could prevent their demise by counterbalancing the pro-apoptotic signals delivered by ITIM-containing FcγRIIb. As IgM-expressing memory B cells have been postulated to be crucial for long-term maintenance of the memory B-cell pool^49^, it will be worthwhile investigating whether IgM^+^ BM plasma cells could fulfill a similar function for the plasma cell component of B-cell memory.

In conclusion, our study provides three novel elements of information: (i) BCR-expressing IgM^+^ plasma cells are fully mature and constitute one of the component of the BM plasma cells compartment generated by immunization, (ii) they can be activated by antigen *in vivo*, (iii) antigen stimulation profoundly remodels their gene expression profile and licences them for cytokine production. Altogether, our findings suggest that BM IgM^+^ plasma cells may represent a previously unrecognized component of the reactive humoral memory.

## Methods

### Mice

C57BL/6J wild-type (WT) mice were purchased from Charles River Laboratories and maintained in pathogen-free conditions at the Plateau de Biologie Expérimentale de la Souris (Ecole Normale Supérieure de Lyon, France). *Blimp*^*gfp*^ mice^17^ were crossed with WT mice to produce *Blimp*^*gfp/+*^ heterozygous mice used in our experiments. *Aicda*^*−/−*^ mice were kindly provided by T. Honjo^50^. Quasi-monoclonal (QM) transgenic mice were kindly provided by Dr M. Cascalho (Department of Surgery and Microbiology and Immunology, University of Michigan Medical School, Ann Arbor, MI 48109, USA). They are hemizygous for a targeted insertion of a rearranged NP-specific V-D-J (VH17.2.25-DSP2.3-JH4) heavy-chain segment and a targeted deletion of the JH region on the other allele and homozygous for deletion of the Jκ loci^51^. The F1 resulting from the crossing of QM mice with *Blimp*^*gfp/+*^ or WT mice were used for Ca^++^ fluxes experiments. *mEGFP/mb-1*^*inv*^ mice have been kindly provided by Pr. Michael Reth (Centre for Biological Signalling Studies BIOSS, Freiburg, Germany). They carry two conditional *mb-1/Ig*α alleles in which *mb-1* was cloned in the opposite transcriptional orientation relative to the enhanced green fluorescent protein (EGFP). This construct directs expression of a membrane-associated form of EGFP but not mb-1/Igα. It is flanked by inverted *loxP* sites that allow switching on Igα expression under the control of the Cre recombinase as previously described^20^. F1 mice originating from the crossing of *mEGFP/mb-1*^*inv*^ mice with Balb/C animals carrying a WT Igα allele (designated as *mb-1/mEGFP* mice) have been used here as conventional Igα reporter mice. IL-10 reporter (Vert-X) mice were purchased from the Jackson Laboratory. All strains of mice were on the C57BL/6J genetic background, except *mEGFP/mb-1*^*inv*^ mice that were on the Balb/C genetic background. They were bred under specific pathogen-free conditions in our animal facility and were used at 8–16 weeks of age. All studies and procedures were performed in accordance with EU guidelines and approved by the local Animal Ethics Evaluation Committee (CECCAPP Lyon, registered by the French National Ethics Committee of Animal Experimentation under no.15). All experiments were conducted with 8- to 12-week old female mice.

### Immunizations

For NP-dextran immunization, mice were injected subcutaneously with 200 μg NP conjugated to dextran (Biosearch Technologies) diluted in sterile and pyrogen-free phosphate-buffered saline (PBS) and with the TLR agonist CpG1668 (5′-TCCATGACGTTCCTGATGCT-3′, MWG Operon, 80 μg per mouse) administered two days after NP-dextran vaccination as described earlier^10^. For NP-KLH immunization, a solution containing 400 μg of NP-KLH (Biosearch Technologies) and 80 μg of CpG 1668 oligonucleotides prepared in pyrogen-free PBS was combined with IFA at a 1:1 (vol/vol) ratio and emulsified immediately before subcutaneous immunization of mice. For antigenic boost of plasma cells *in vivo*, mice were injected intravenously with 200 μg of NP-dextran or NP-KLH, diluted in 200 μl PBS.

### Cell preparation and *ex vivo* stimulation

Single cells suspensions from spleen were obtained by mechanical dissociation in RPMI medium (GIBCO, Life Technologies) containing 10% FCS (HyClone, ThermoFisher). BM cells were obtained by flushing tibias and femurs with the same medium. To prepare enriched BM plasma cells populations, BM cells were stained with an APC-conjugated anti-CD138-mAb (BD Biosciences) then incubated with anti-APC MicroBeads and positively selected through isolation with LS columns exposed to a strong magnetic field (Miltenyi Biotec). Antigen and surrogate antigen used for *in vitro* stimulation of B cells and plasma cells were used at the following concentrations: (i) NP-dextran and NP-KLH at 15 μg ml^−1^, (ii) goat anti-mouse IgM F(ab′)2 Abs (Jackson ImmunoResearch), goat anti-mouse-Ig (G+A+M) F(ab′)2 Abs (Southern Biotech) and goat-anti-human lymphotactin (negative control, Abcam) at 10 μg ml^−1^, (iii) ionomycin (Sigma) at 1 μg ml^−1^.

### Flow cytometry analysis

Before staining, Fcγ receptors were blocked for 15 min at 4 °C with 2.4. G2 hybridoma supernatant. Antibodies with the following specificities and conjugations were purchased from the following manufacturers. The clone name and dilution used are indicated in brackets after the antibody target. BD Biosciences: CD3ɛPE-Cy7 (145-2C11, 1/200), CD11b-PerCP-Cy5.5 (M1/70, 1/200), CD19-PerCP-Cy5.5 (1D3, 1/200), CD22-BUV 395 (Cy34.1, 1/200), CD45R/B220 conjugated to APC-H7 or BV786 (RA3-6B2, 1/200), CD69-APC (H1.2F3, 1/200), CD79a-FITC (F11-172, 1/100), CD138 conjugated to APC, BV605 or BV711 (281-2, 1/200), IgM-PerCP-Cy5.5 (R6-60.2, 1/50), IgD-BV510 (11-26c.2a, 1/200), IgG1-BV421 (A85-1, 1/250), IA/IE-PE-CF594 (M5/114, 1/400), Ki67-APC (B56, 1/100), Lamp-1-PE (ID4B, 1/200), Ly6C-PE-Cy7 (AL-21, 1/200), p-Syk-AF647 (17A/P-ZAP70, 1/10), p-Blnk-AF647 (pY84, 1/10), p-ERK-BV421 (20A, 1/10), Pax5-BV421 (1H9, 1/200), Ly6G-APC (1A8, 1/200). The FITC-conjugated anti-CD79b (HM79-12, 1/100), APC-conjugated anti-Igλ light-chain (RML-42, 1/200) and BV421-conjugated anti-CD20 (SA275A11, 1/200) mAbs were purchased from Biolegend. To visualize NP-binding cells, cells were incubated for 15 min at +4 °C with a 4 μg ml^−1^ solution of a the NP hapten conjugated to PE (purchased from Biosearch Technologies) prepared in staining buffer. The Cytofix/Cytoperm kit (BD) was used to fix and permeabilize cells for intracellular stainings. Analysis of Pax5, Blimp-1 and CD19 expression (Fig. 2) by PB and plasma cells was conducted on CD138-enriched cells isolated from the spleen (at day 7 post-immunization) and from the BM (at day 45 post-immunization) of *Blimp*^*gfp/+*^ mice, respectively. Non-B and non-plasma cells used as negative staining controls were gated as GFP^−^/CD19^−^/B220^−^ cells in *Blimp*^*gfp/+*^ mice (Fig. 2) or as CD138^−^/CD19^−^/B220^−^ cells in *mb-1/mEGFP* mice (Fig. 4c). Data were collected using LSRII and LSR Fortessa flow cytometers (BD) and analysed with FlowJo software (Tree Star).

Plasma cells numbers recovered in the positive fraction after magnetic selection of bone marrow mononuclear cell (MNC) with anti-CD138 Abs represented on average 70% of the plasma cells numbers present in the starting unfractionated bone marrow MNC population. We thus assumed that about 70% of the bone marrow NP-specific plasma cells population was captured by the enrichment procedure and we took this correction factor into account for calculation of the overall numbers of NP-specific plasma cells in the bone marrow. This calculation was based on the assumption that the marrow cells located in two femurs represent 12.6% of the total bone marrow^52^.

### Phospho flow analysis

Splenocytes recovered from QM X C57Bl/6 F1 mice were used for analysis of Syk and Blnk phosphorylation following *in vitro* stimulation of NP-specific B cells with NP-dextran or NP-KLH. Enriched BM plasma cells populations prepared by positive CD138 selection from immunized *Blimp*^*gfp/+*^ mice were used for analysis of Syk and Blnk phosphorylation in TI or TD BM plasma cells (Fig. 4). Cells were stimulated *ex vivo* with antigen (NP-dextran, NP-KLH) or surrogate antigen (anti-Ig Abs) for 3 min then fixed using the PerFix EXPOSE kit (Beckman Coulter) before staining with anti-p-Syk and anti-p-Blnk mAbs, together with NP-PE, anti-B220 and anti-CD19 mAbs (for B cell samples). NP-binding (NP^+^) and non NP-binding (NP^−^) B cells were gated as B220^+^/CD19^+^/NP-PE^+^ and B220^+^/CD19^+^/NP-PE^−^ cells, respectively. NP-binding and non NP-binding plasma cells were gated as GFP^+^/CD138^+^/NP-PE^+^ and GFP^+^/CD138^+^/NP-PE^−^ cells respectively. DAPI (Molecular Probes) was used to exclude dead cells. The delta MFI values used to quantitate the level of Syk and Blnk phosphorylation in Figs 4e and 5c,e,h were calculated by subtracting the MFI values of the staining histograms for unstimulated cells from those of stimulated cells.

### Epifluorescence microscopy in flow

Flash red-conjugated streptavidin polystyrene microspheres of 400 nM diameter (Bangs Laboratories) were coated with biotinylated NP-OVA (BioSearch). Enriched BM plasma cells populations recovered 45 days after immunization of *Blimp*^*gfp/+*^ mice with NP-dextran were incubated 30 min with NP-OVA-coated beads at a ratio of 30 beads per cell. Cells were then fixed and stained for Lamp-1, and p-ERK using the PerFix EXPOSE kit (Beckman Coulter) and analysed by epifluorescence microscopy on the Amnis (ImageStream X).

### Intracellular Ca^++^ mobilization

Total splenocytes from QM x WT F1 mice and enriched BM plasma cells from QM x *Blimp*^*gfp/+*^ F1 mice immunized 30 days before by NP-dextran were used for these experiments. Cells were stained with a PerCP-Cy5.5-conjugated anti-CD19 mAb (splenocytes) and NP-PE (splenocytes and enriched BM plasma cells) before loading with Indo-1 acetoxymethyl (Indo-1 AM, Invitrogen). Low concentrations of NP-PE were used for staining to limit BCR-mediated signalling at this step. Cells were next resuspended at 6 × 10^6^ cells per ml in 1 ml RMPI medium containing 0.2% BSA (Sigma) and loaded with 1 μM Indo-1 at 37 °C for 45 min. After washing in PBS, cells were maintained in protein-free RPMI at 37 °C. After a baseline was established, cells were stimulated with: NP-dextran, goat anti-mouse IgM F(ab′)2 Abs, goat-anti-human lymphotactin F(ab′)2 Abs (negative control) or ionomycin. Ca^++^ mobilization was analysed on the LSRII analyzer in: (i) NP-binding B cells (CD19^+^/NP-PE^+^) and plasma cells (GFP^+^/NP-PE^+^) and (ii) non NP-binding B cells (CD19^+^/NP-PE^−^) and plasma cells (GFP^+^/NP-PE^−^). Ca^++^ flux was determined by measurement of the fluorescence ratio of 440/40 nm (calcium-bound Indo-1) to 530/30 nm emissions (calcium-free Indo-1), i.e., the violet to blue ratio.

### BrdU incorporation and staining

Mice were given BrdU in the drinking water (1 mg ml^−1^, Sigma) from day 0 to day 8 after immunization with NP-dextran (pulse period) after which BrdU was withdrawn (chase period). BrdU incorporation was determined using the BrdU Flow Kit (BD Biosciences) according to the manufacturer's instructions.

### ELISPOT assays

Cells were added at various concentrations into Multiscreen HTS plates (Millipore) previously coated with NP-BSA (to enumerate NP-specific Ab-secreting cells/ASCs) or anti-mouse kappa and lambda light chain (to enumerate IgM, IgG and IgA-producing ASCs). After overnight incubation, spots were revealed with alkaline-phosphatase-conjugated: anti-mouse kappa and lambda light chains (to reveal all heavy chain isotypes) or anti-mouse IgM, IgG2b, IgG2c or IgG3 (Southern Biotechnology Associates) to reveal Abs of the corresponding subclasses. Plates were developed with BCIP/NBT substrate (Sigma). Spots were counted and analysed with the ImmunoSpot Analyser S6 Ultra-V (CTL-Europe GmbH, Bonn, Germany).

### RNA isolation and quantitative real-time RT-PCR

Total RNA from 50 to 500 sorted plasma cells was reverse-transcribed then amplified using the CellAmp Whole Transcriptome Amplification Kit (Ozyme) according to the manufacturer's protocol. The reaction mix was diluted 1/10 and stored at −20 °C until real-time PCR analysis. Specific primer sets for the murine membrane-bound or secreted IgM, IgG2b, IgG2c and IgG3 heavy chains transcripts were designed using the Primers3 software and were purchased from Invitrogen Life Technologies. The real-time PCR was performed on an Applied Biosystems PRISM 7000 using the SYBR Green Master Mix: SYBR Premix Ex Taq II (Tli RNase H Plus) kit (Ozyme) according to the manufacturer's instructions. The relative quantity of each transcript was normalized according to the expression of the housekeeping gene GAPDH. The primer sequences (forward/reverse) used are shown in Supplementary Table 1.

### Microarray analysis

*Blimp*^*gfp/+*^ mice were immunized subcutaneously with 200 μg NP-dextran (day 0) and CpG (day 2) as described above. They were boosted intravenously 60 days later with 200 μg NP-dextran (experimental group) or injected with PBS (control group). Twelve hours later, bone marrow cells were harvested, enriched for plasma cells by CD138 positive selection and stained with NP-PE. NP-specific plasma cells were gated as CD138^+^/GFP^+^/NP-PE^+^ cells. 1,000 to 5,000 NP-specific plasma cells were directly sorted on a FACSAria sorter (BD) into RNAprotect Cell Reagent (Qiagen).

After pelleting, the RNAprotect buffer was replaced by RLT Plus buffer (Qiagen) and the samples were homogenized by vortexing for 1 min. Genomic DNA contamination was removed using gDNA Eliminator spin columns (Qiagen). After addition of ethanol, the samples were applied to RNeasy MinElute spin columns (Qiagen) followed by several washing steps. Finally total RNA was eluted in 12 μl of nuclease-free water. Purity and integrity of the RNA was assessed on the Agilent 2100 Bioanalyzer with the RNA 6000 Pico LabChip reagent set (Agilent).

One to 5 ng of total RNA was reverse transcribed into double-stranded cDNA in a two-step process, introducing a SPIA tag sequence. Bead-purified cDNA was amplified by a SPIA amplification reaction followed by an additional bead purification. Three microgram of SPIA cDNA were fragmented, terminally biotin-labelled and hybridized to an Affymetrix Mouse Genome 430 PM 16-Array Plate. An Affymetrix GeneTitan system (Affymetrix, Inc., Santa Clara, CA, USA) was used for hybridization, washing, staining and scanning. RNA extraction and sample processing were performed at an Affymetrix Service Provider and Core Facility: the KFB Center of Excellence for Fluorescent Bioanalytics (Regensburg, Germany; www.kfb-regensburg.de). Affymetrix CEL files were analysed in R using the Bioconductor suite of packages. Raw probe signals were background-corrected using the maximum likelihood estimation of the normal-exponential mixture model and normalized using the variance stabilization normalization, followed by quantile normalization. For summarization, probe signals for each probe set of each sample were summarized into a single value using the summarization step of the Robust Multichip Average (RMA) approach. Non-informative genes were filtered using the I/NI algorithm. Linear models were applied using the limma package to compute the mean expression level for each cell type. Statistical contrasts were then applied to compute differential expression between the different cell types. The empirical Bayes method was used to compute moderated *P*-values that were then corrected for multiple comparisons using the Benjamini and Hochberg's false discovery rate (FDR) controlling procedure. CEL files analysis was performed by AltraBio (Lyon, France; www.altrabio.com).

### Statistics

GraphPad Prism software was used for statistical analysis. The Mann–Whitney non-parametric test was applied throughout the study to compare experimental groups. For bar graphs, data are expressed as means±s.d.s, as indicated in the figure legends. Results were considered statistically significant when *P* value <0.05 (*P<0.05, **P<0.01 and ***P<0.001).

### Data availability

The microarray data that support the findings of this study have been deposited in Gene Expression Omnibus with the primary accession code GSE80216. The authors declare that all other data supporting the findings of this study are available within the article and its Supplementary Information Files, or are available on request from the corresponding author.

## Additional information

**How to cite this article:** Blanc, P. *et al*. Mature IgM-expressing plasma cells sense antigen and develop competence for cytokine production upon antigenic challenge. *Nat. Commun.* **7,** 13600 doi: 10.1038/ncomms13600 (2016).

**Publisher's note**: Springer Nature remains neutral with regard to jurisdictional claims in published maps and institutional affiliations.

## Supplementary Material

Supplementary Information Supplementary Figure 1 and Supplementary Table 1

Supplementary Data 1 Detailed information on the 1000 most differentially expressed genes between unstimulated and Ag-stimulated NP-specific IgM+ bone marrow plasma cells.

## Figures and Tables

**Table 1:** Differentially expressed genes in the antigen-stimulated IgM^+^ plasma cell group as compared with the unstimulated IgM^+^ plasma cell group.

**Gene symbol**	**Gene name**	**FC**	***q*** **value**
*Differentially expressed genes, upregulated (q<0.05)*			
***Ccl5***	**Chemokine (C-Cmotif) ligand 5**	**8.6**	**0.0014**
***Fxyd5***	**FXYD domain-containing ion transport regulator 5**	**3.1**	**2.9e−5**
***Wnt10a***	**Wingless related MMTV integration site 10a**	**3.1**	**4.9e−7**
***Fah***	**Fumarylacetoacetate hydrolase**	**2.6**	**0.0014**
***Slfn2***	**Schlafen 2**	**2.5**	**0.0130**
***Irf8***	**Interferon regulatory factor 8**	**2.5**	**0.0095**
***Siggir***	**Single immunoglobulin and toll-interleukin 1 receptor TIR domain**	**2.5**	**0.002**
***Serpinc1***	**Serine (or cystein) peptidase inhibitor, clade C, member 1**	**2.4**	**0.02**
***Bhlhe40***	**Basic helix-loop-helix family, member e40**	**2.3**	**0.049**
***Grap***	**GRB2-related adaptor protein**	**2.2**	**0.017**
***Ctla4***	**Cytotoxic T-lymphocyte-associated protein 4**	**2.2**	**0.047**
***Lsp1***	**Lymphocyte specific 1**	**2.2**	**0.015**
***Ptprcap***	**Protein tyrosine phosphatase receptor type C-associated protein**	**2.1**	**0.037**
***Srgn***	**Serglycin**	**2.1**	**6.7e−8**
***Gpnmb***	**Glycoprotein (transmembrane) nmb**	**2.1**	**0.042**
***Ddt***	**D-dopachrome tautomerase**	**2**	**9.3e−4**
***Endod1***	**Endonuclease domain-containing 1**	**2**	**0.015**
***Tg***	**Thyroglobulin**	**2**	**0.042**
***Casp4***	**Caspase 4**	**2**	**0.018**
			
*Differentially expressed genes, downregulated (q<0.05)*			
***Reln***	**reelin**	**0.47**	**0.018**
*Negr1*	Neuronal growth regulator 1	0.51	0.022
*Plekhm3*	Pleckstrin homology domain-containing family M, member 3	0.54	0.017
*Pfkm*	Phosphofructokinase, muscle	0.55	0.027
*C1qtnf1*	Complement C1q tumour necrosis factor-related protein 1	0.55	0.049
*Gpr155*	G protein-coupled receptor 155	0.56	0.0012
*Upb1*	Ureidopropionase, beta	0.58	0.038
*Pgam2*	Phosphoglycerate mutase 2	0.60	0.034
*Dhx33*	DEAH box polypeptide 33	0.60	0.021
*Tnfrsf17*	Tumour necrosis factor receptor superfamily	0.60	0.002

FC, fold change.

Only genes with a fold change superior or equal to 2 (for upregulated genes) or with a fold change inferior or equal to 0.60 (/1.7) (for downregulated genes) have been included in the table. The 20 most significantly modulated genes (highest absolute fold change) in the antigen-stimulated plasma cells group are shown in bold characters. They constitute the study group that has been analysed by MGSA (Table 2).

**Table 2:** Gene Ontology terms most strongly associated with the 20 most significantly modulated genes in the antigen-stimulated IgM^+^ plasma cell group.

	**Description**	**Identity**	**Freq.**	**Genes**	**Nb**
**1**	**Regulation of cytokine production**	**GO:0001817**	**0.32**	***Ccl5, Irf8, Siggir, Srgn, Casp 4***	**349**
**2**	**Cytokine production**	**GO:0001816**	**0.21**	***Ccl5, Irf8, Siggir, Srgn, Casp 4***	**391**
3	Thyroid hormone metabolic process	GO:0042403	0.12	*Reln, Tg*	15
4	Cellular amino acid metabolic process	GO:0006520	0.11	*Fah, Reln, Tg*	213
5	Phenol-containing compound metabolic process	GO:0018958	0.057	*Reln, Tg*	59
6	Negative regulation of sequence-specific DNA binding transcription factor activity	GO:0043433	0.044	*Sigirr, Bhlhe40*	114
7	Cytokine secretion	GO:0050663	0.042	*Srgn, Casp4*	86
8	Regulation of cytokine secretion	GO:0050707	0.042	*Srgn, Casp4*	75
9	Entrainment of circadian clock by photoperiod	GO:0043153	0.039	*Bhlhe40*	5

Freq. is the frequency with which the gene set is activated in the algorithm; Nb, numbers of genes in the reference set.

Data have been analysed with the MGSA (Model-based Gene Set Analysis) algorithm. The study gene set encompasses the 20 genes with the highest absolute fold change (and adjusted *P* value<0.05) in the antigen-stimulated plasma cell group. MGSA infers the ‘active' gene sets of the experiment, among all considered gene sets, given the actual genes observed as differentially expressed. The column entitled ‘genes' details the genes that are shared by the study gene set and by the gene set defining the GO term. Bold identifies biological processes that are further discussed in the manuscript.
