# Supplementary material for: Mature IgM-expressing plasma cells sense antigen and develop competence for cytokine production upon antigenic challenge
Source: Nat Commun. 2016 Dec 7;7:13600. doi: 10.1038/ncomms13600 (PMC5150646; doi:10.1038/ncomms13600)
Supplement: Supplementary Information — Supplementary Figure 1 and Supplementary Table 1 [file ncomms13600-s1.pdf]

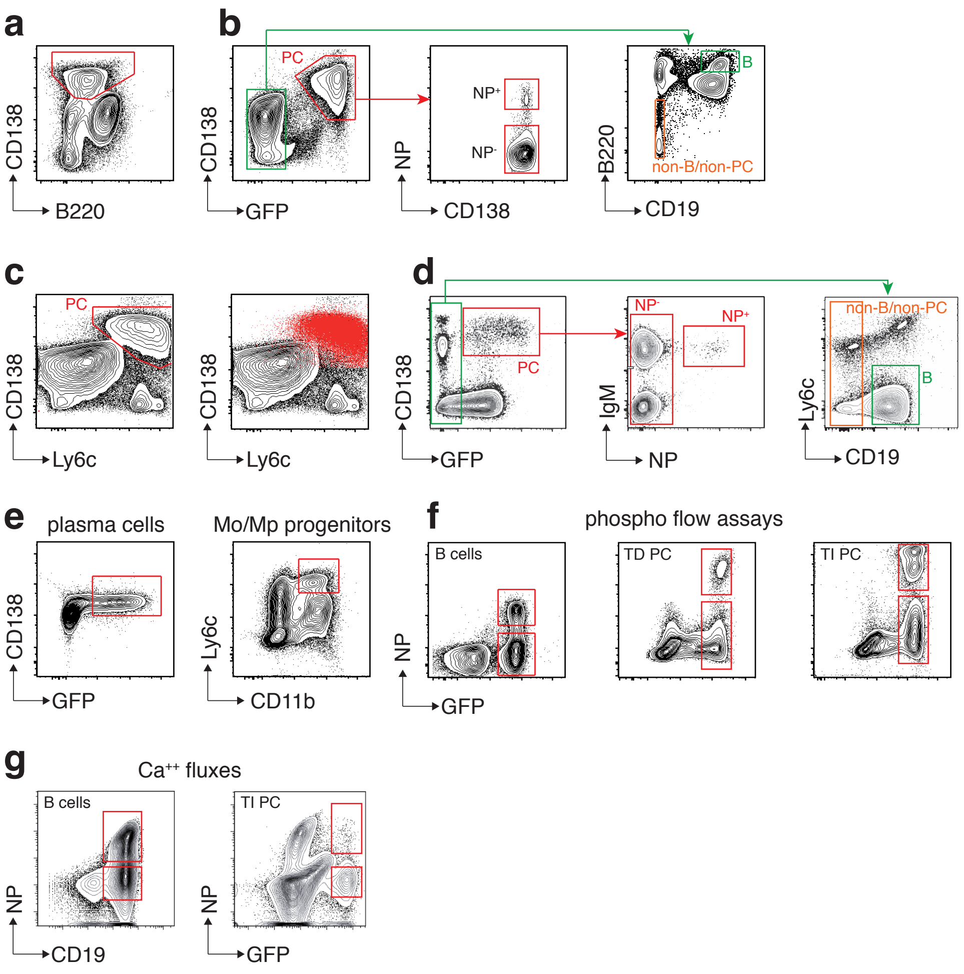

a
b
c
d
e
f
g
CD138
B220
PC
GFP
NP
NP+
NP-
CD138
B220
CD19
B
non-B/non-PC
Ly6c
IgM
plasma cells
Mo/Mp progenitors
CD11b
phospho flow assays
B cells
TD PC
TI PC
Ca++ fluxes

Supplementary Figure 1. FACS gating schemes. (**a**) Representative FACS pre-gating of BM PC for experiments displayed in Fig. 1a. (**b**) Representative FACS gatings for polyclonal PC, NP-specific PC, mature BM B cells and non-B/non-PC (non B) cells in *Blimp*$^{gfp/+}$ mice. **(c)** Back-gating of GFP^{+}/CD138^{hi} PC (in red) on the Ly6c/CD138 staining profile of enriched PC populations of *Blimp*$^{gfp/+}$ mice. **(d)** Representative FACS gatings for polyclonal PC, NP-specific PC, mature BM B cells and non-B/non-PC (non B) cells in Igα reporter mice. (**e**) Representative FACS gatings of BM PC (CD138^{+}/GFP^{+}) and Mo/Mp progenitors (Ly6c^{hi}/CD11b^{+}) for the BrdU and Ki67 experiments shown in Fig. 3. (**f**) FACS gating of NP-binding and non NP-binding splenic B cells (from QM X C57Bl/6 F1 mice) and BM PC (from *Blimp*$^{gfp/+}$ mice) for the phospho flow assays shown in Fig. 4d and 5b. (**g**) FACS gating of NP-binding and non NP-binding splenic B cells (from QM X C57Bl/6 F1 mice) and BM TI PC (from QM X *Blimp*$^{gfp/+}$ mice) for analysis of Ca^{++} mobilization. All FACS gatings shown were realized on enriched PC populations obtained after magnetic positive selection of BM MNC with an anti-CD138 mAb.

Supplementary Table 1. Sequences of the primers used for quantitative real-time RT-PCR analysis.

	Forward primer	Reverse primer
**mIgM**	5'-CCCCAGGCTTCTACTTTACCC-3'	3'-CTTCCTCCTCAGCATTCACC-5'
**mIgG2b**	5'-GTGAGACACGAGGGTCTGAA-3'	3'-CGTCCAGCTCCCCATCCT-5'
**mIgG2c**	5'-CACGAGGGTCTGCACAATCA-3'	3'-CGTCCAGCTCCCCATCCT-5'
**mIgG3**	5'-GGCTCTCCATAACCACCACA-3'	3'-CGTCCAGCTCCCCATCCT-5'
**sIgM**	5'-TGGAACTCCGGAGAGACCTA-3'	3'-TCAGACATGATCAGGGAGACA-5'
**sIgG2b**	5'-GTGAGACACGAGGGTCTGAA-3'	3'-ACCTGAGAGCTTTGTGGGTG-5'
**sIgG2c**	5'-CACGAGGGTCTGCACAATCA-3'	3'-GACCCAGGAGCATTGTGTGT-5'
**sIgG3**	5'-GGCTCTCCATAACCACCACA-3'	3'-TGTAAGACCCGAGGAATGGC-5
**GAPDH**	5'-GCATGGCCTTCCGTGTCC-3'	3'-TGTCATCATACTTGGCAGGTTTCT-5'

The primers for the membrane and secreted forms of Igs are designated by the letters m and s, respectively, preceding designation of the Ig isotype.
